# Supplementary material for: Aerosol effects on clouds are concealed by natural cloud heterogeneity and satellite retrieval errors
Source: Nat Commun. 2022 Nov 30;13:7357. doi: 10.1038/s41467-022-34948-5 (PMC9708656; doi:10.1038/s41467-022-34948-5)
Supplement: Supplementary file 1 — Supplementary Information [file 41467_2022_34948_MOESM1_ESM.pdf]

Supplementary Material - Aerosol effects on clouds are concealed by natural cloud heterogeneity and satellite retrieval errors

Antti Arola[1,*], Antti Lipponen[1], Pekka Kolmonen[1], Timo H. Virtanen[1], Nicolas Bellouin[2], Daniel P. Grosvenor[3], Edward Gryspeerdt[4], Johannes Quaas[5], and Harri Kokkola[1]

[1]Finnish Meteorological Institute, Finland.
[2]Department of Meteorology, University of Reading, Reading, UK
[3]School of Earth and Environment, University of Leeds, Leeds, UK
[4]Space and Atmospheric Physics Group, Imperial College London, UK
[5]Institute for Meteorology, Universität Leipzig, Leipzig, Germany
[*]Corresponding author: antti.arola@fmi.fi

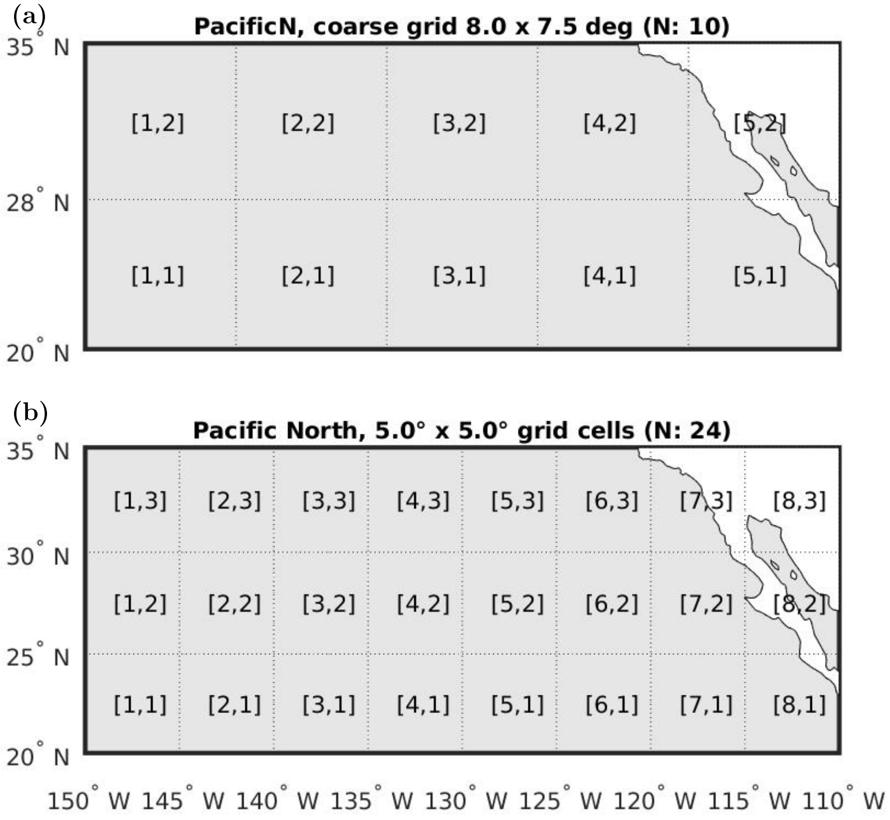

Supplementary Figure 1: **Division of the Pacific North area into smaller grid cells.** The indexing is used in the scatter plots below. (a) $8° \times 7.5°$ grid cells. (b) $5° \times 5°$ grid cells.

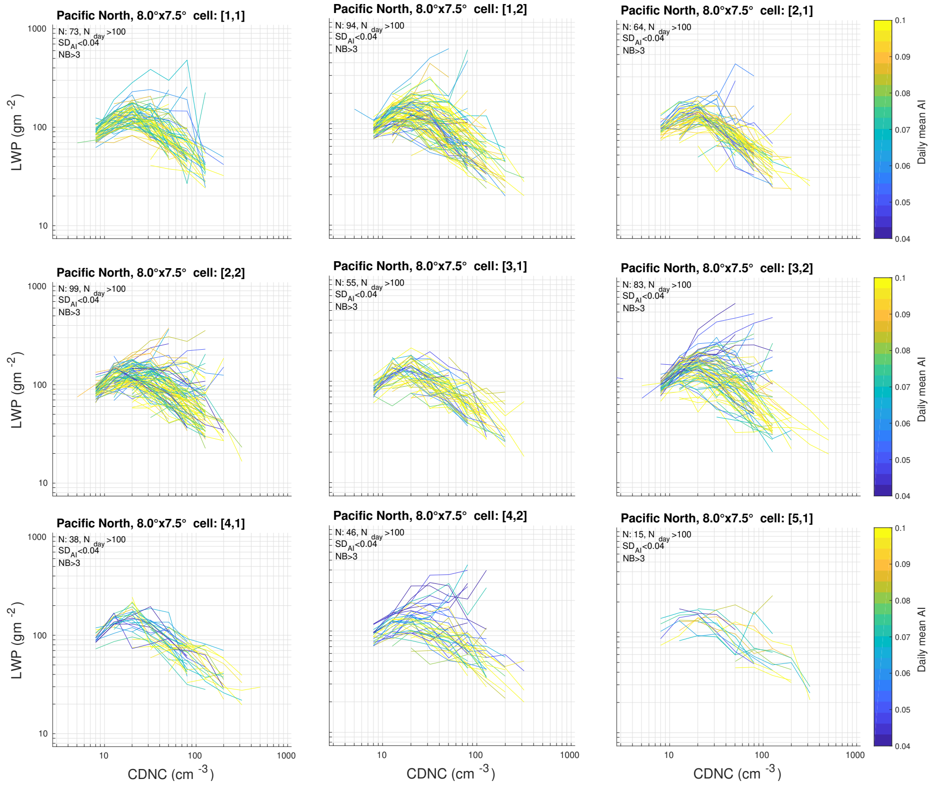

Supplementary Figure 2: **Liquid water path (LWP) vs cloud droplet number concentration (CDNC) as daily binned mean plots for** $8^\circ \times 7.5^\circ$ **grid cells.** The plots are similar to Figure 1c in the article, but for the Pacific North area divided into ten $8^\circ \times 7.5^\circ$ grid cells. N shows the number of days in the plot and only those days are shown for which the number of data points N_{day} exceeds 100. Only those points are shown for which the number of data points per CDNC (NB) exceeds 3. The daily standard deviation of the Aerosol Index (SD_{AI}) is limited to below 0.04 in order to limit the impact of aerosol changes on the observed relationships. Lines are coloured by the area mean AI. The indexes in square brackets refer to the grid cells defined in the map shown in Supplementary Figure 1a. One of the cells did not contain sufficient data for plotting.

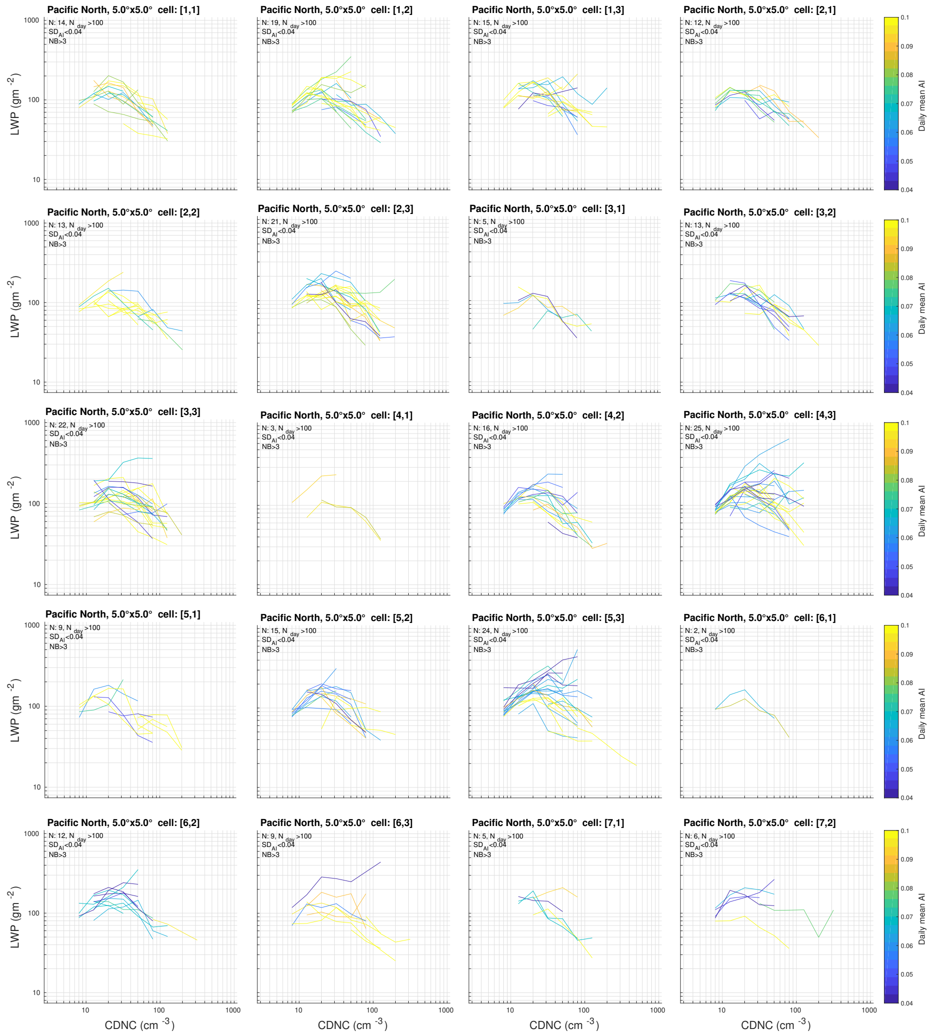

Supplementary Figure 3: **Liquid water path (LWP) vs cloud droplet number concentration (CDNC) as daily binned mean plots for $5° \times 5°$ grid cells.** Same as Supplementary Figure 2 above, with area divided into 24 $5° \times 5°$ grid cells. Four cells did not contain sufficient data for plotting. Figure 1c in the article corresponds to grid cell [3,2].

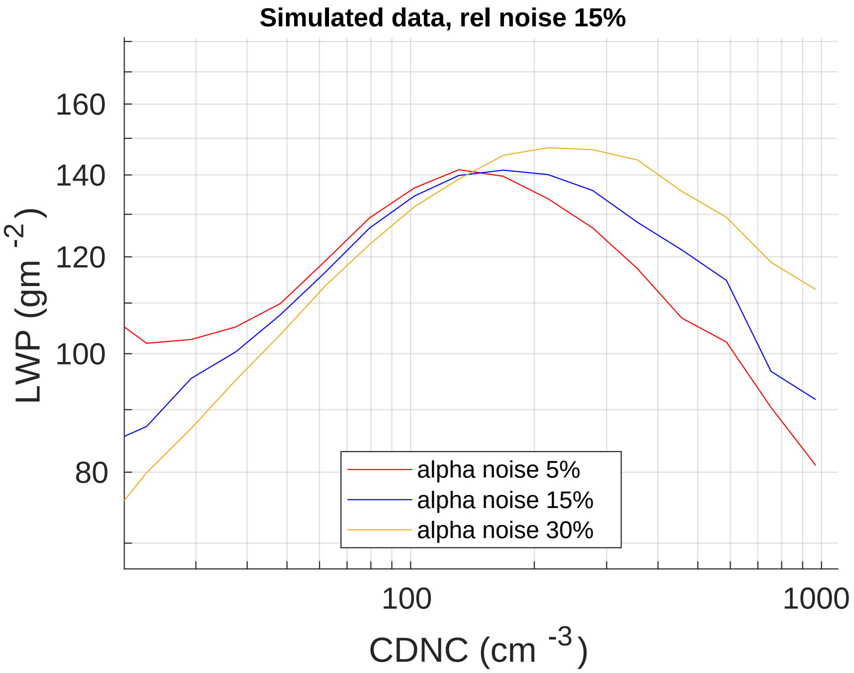

Supplementary Figure 4: **Median values of liquid water path (LWP) as a function of cloud droplet number concentration (CDNC), resulting from varying levels of relative error in α.** The α-term in the Equation 1 (in the main text) relates CNDC to cloud optical depth (COD) and cloud effective radius (CER) and includes the adiabatic factor and condensation rate correction. Errors of both COD and CER were 15% in this simulation.

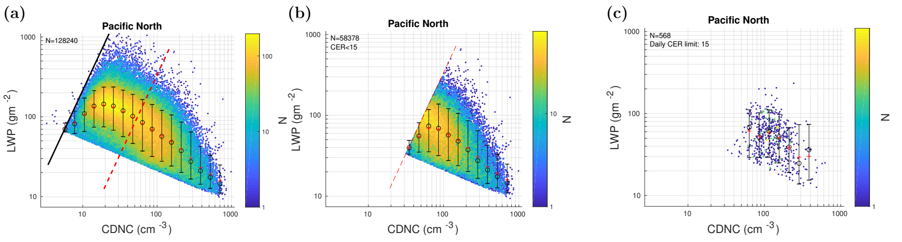

Supplementary Figure 5: **Effect of cloud effective radius (CER) thresholds on liquid water path (LWP) vs cloud droplet number concentration (CDNC) plots.** (a) All data are included with the cloud effective radius of CER=30μm and CER=15μm shown by black and dashed red line, respectively. (b) Only data with CER less than 15μm are included. (c) A more stringent CER=15μm threshold was applied as follows: if there was any case of CER larger than 15μm in the entire Pacific North area in a given day, then the full day was entirely excluded.

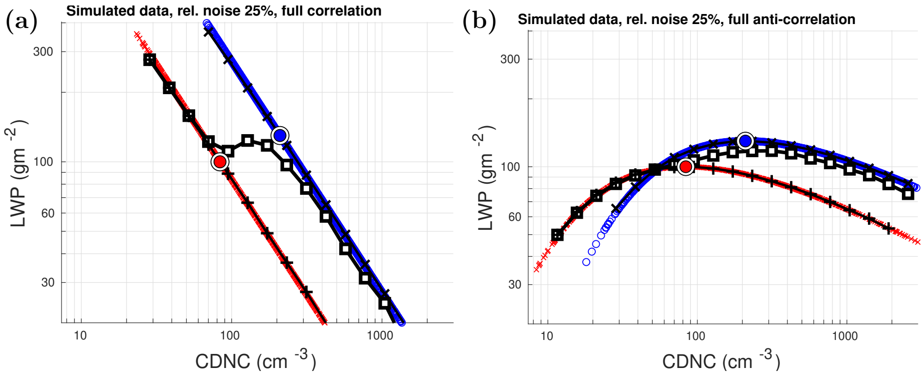

Supplementary Figure 6: **Effect of correlation on the simulated relation between liquid water path (LWP) and cloud droplet number concentration (CDNC) for less and more polluted cases.** Same as Figure 4 in the article, but now with (a) fully correlated cloud effective radius (CER)/cloud optical depth (COD) error, and (b) fully anti-correlated error.

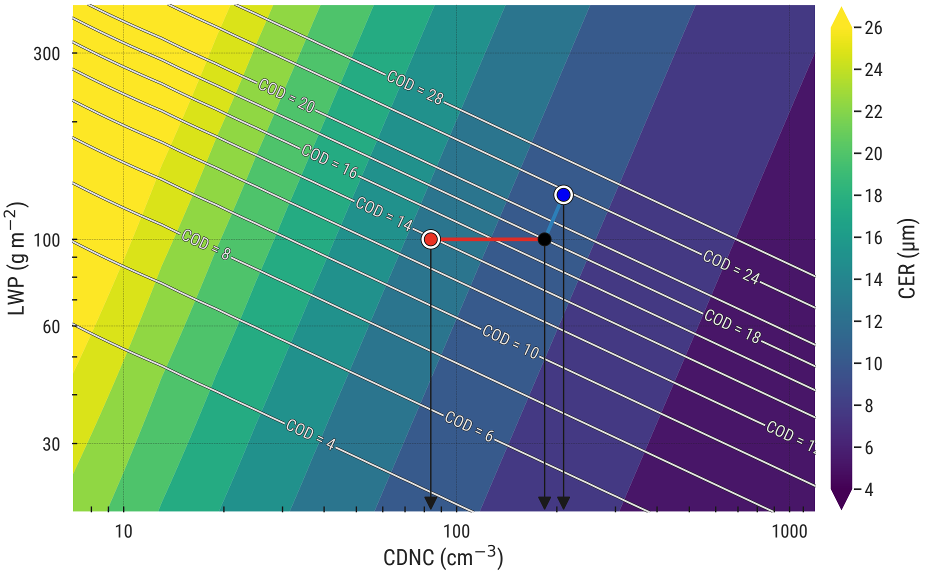

Supplementary Figure 7: **Cloud droplet number concentration (CDNC) - liquid water path (LWP) relationships as a function of cloud effective radius (CER) and cloud optical depth (COD).** Calculated by using Equations 1 and 2 (in the main text). Red and blue circles can be taken to illustrate the mean CDNC and LWP of less and more polluted case of Figure 4 in the main text. Black circle shows the corresponding change in CDNC if the LWP is constant.

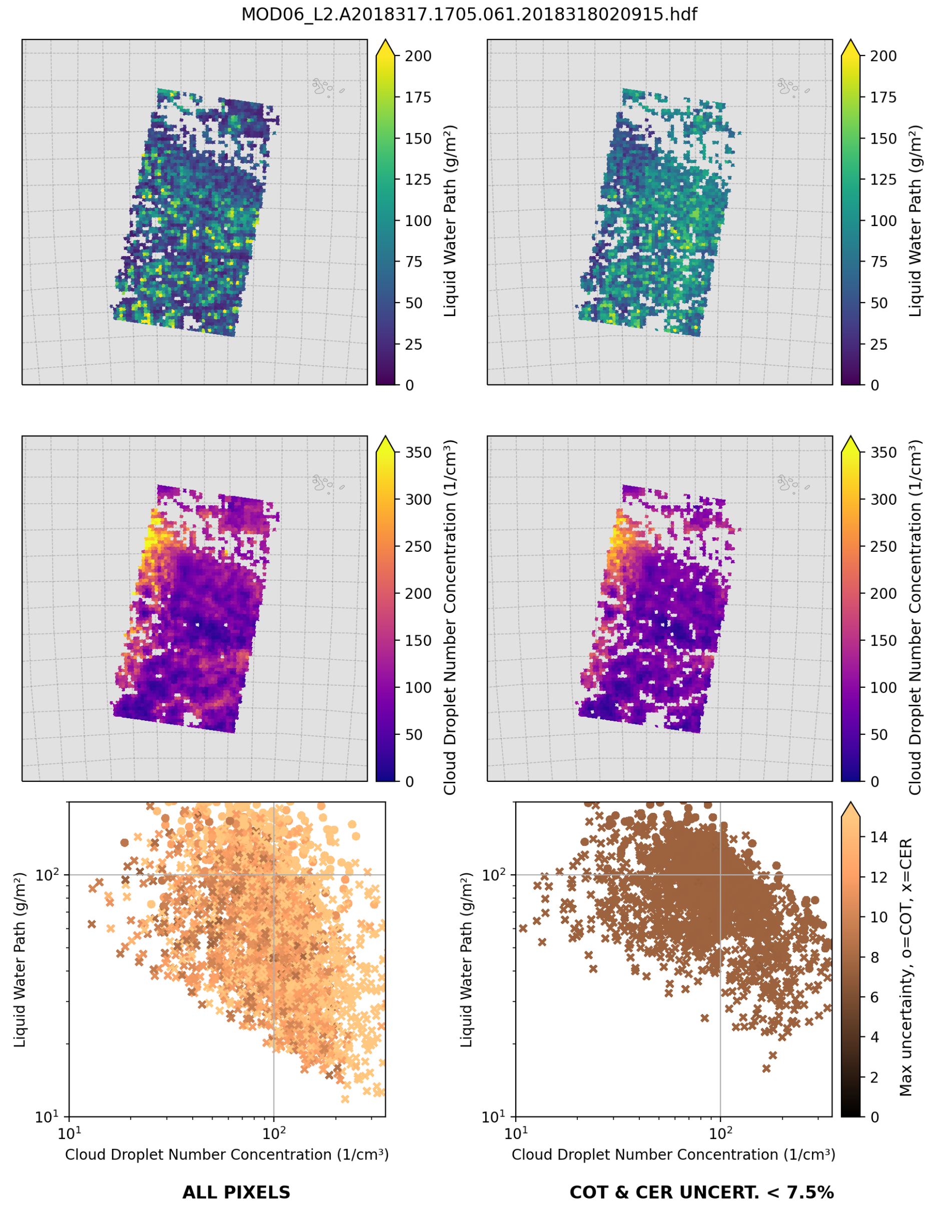

Supplementary Figure 8: **Liquid water path (LWP) and cloud droplet number concentration (CDNC) from single satellite overpass.** LWP (upper panel) and CDNC (middle panel) aggregated to $0.25^{\circ} \times 0.25^{\circ}$, over South Pacific region, November 14th 2018. The lowest panel shows LWP distribution as a function of CDNC corresponding to these spatial LWP and CDNC fields. All the data are shown in the left-hand-side column (applying the filters of Gryspeerdt et al.[1]), while in the right-hand-side column shows such a subset when only cloud optical depth (COD) and cloud effective radius (CER)s measurements with uncertainty less than 7.5% were included.

Supplementary References

[1] Gryspeerdt, E., Goren, T., Sourdeval, O., Quaas, J., Mülmenstädt, J., Dipu, S., Unglaub, C., Gettelman, A., and Christensen, M.: Constraining the aerosol influence on cloud liquid water path, Atmos. Chem. Phys., 19, 5331–5347, https://doi.org/10.5194/acp-19-5331-2019, 2019.
